# Perceptual Averaging in Individuals with Autism Spectrum Disorder

**DOI:** 10.3389/fpsyg.2016.01735

**Published:** 2016-11-07

**Authors:** Jennifer E. Corbett, Paola Venuti, David Melcher

**Affiliations:** ^1^Aysel Sabuncu Brain Research Center, Bilkent UniversityAnkara, Turkey; ^2^Department of Cognitive Science and Education, University of TrentoTrento, Italy; ^3^Center for Mind/Brain Sciences, University of TrentoTrento, Italy

**Keywords:** perceptual averaging, ASD, mean size, perceptual adaptation, vision, attention

## Abstract

There is mounting evidence that observers rely on statistical summaries of visual information to maintain stable and coherent perception. Sensitivity to the mean (or other prototypical value) of a visual feature (e.g., mean size) appears to be a pervasive process in human visual perception. Previous studies in individuals diagnosed with Autism Spectrum Disorder (ASD) have uncovered characteristic patterns of visual processing that suggest they may rely more on enhanced local representations of individual objects instead of computing such perceptual averages. To further explore the fundamental nature of abstract statistical representation in visual perception, we investigated perceptual averaging of mean size in a group of 12 high-functioning individuals diagnosed with ASD using simplified versions of two identification and adaptation tasks that elicited characteristic perceptual averaging effects in a control group of neurotypical participants. In Experiment 1, participants performed with above chance accuracy in recalling the mean size of a set of circles (*mean task*) despite poor accuracy in recalling individual circle sizes (*member task*). In Experiment 2, their judgments of single circle size were biased by mean size adaptation. Overall, these results suggest that individuals with ASD perceptually average information about sets of objects in the surrounding environment. Our results underscore the fundamental nature of perceptual averaging in vision, and further our understanding of how autistic individuals make sense of the external environment.

## Introduction

The natural environment is typically complex and full of information, but our perceptual systems have a limited capacity to represent only a fraction of this information in detail at any given moment. One strategy that has been suggested to deal with this fundamental challenge is for the brain to create meaningful statistical summaries of sets of objects from the mass of incoming information in each glance without the need to explicitly encode individual items (for review, see Ariely, 2001; Alvarez, 2011; Corbett and Melcher, 2014a,b). For example, we are able to quickly represent the average size of trees in a forest but unable to represent the sizes of more than a few individual trees in the same forest simultaneously.

This seemingly fundamental process of perceptual averaging is pervasive in visual perception across task demands (e.g., Corbett et al., 2012; Oriet and Brand, 2013), focused attentional constraints (e.g., Chong and Treisman, 2005), and spatial reference frames (Corbett and Melcher, 2014a), and does not rely on precise representations of individual objects (e.g., Parkes et al., 2001; Choo and Franconeri, 2010; Corbett and Oriet, 2011). Average representations of size (Corbett et al., 2012), motion direction (e.g., Anstis et al., 1998), orientation (e.g., Gibson and Radner, 1937), texture density (e.g., Durgin, 1995), and numerosity (Burr and Ross, 2008) also undergo perceptual adaptation, a process by which the extended presentation of a stimulus biases the perception of subsequently presented stimuli in opposite directions along fundamental dimensions of the adapting stimulus. These findings converge to suggest that perceptual averages are encoded as basic features of visual information (Gibson, 1937; Campbell and Robson, 1968; Thompson and Burr, 2009). Furthermore, when the average properties of background information remain stable over time, observers are better able to find salient targets, suggesting that perceptually averaging information allows the visual system to capitalize on the statistical redundancy in the external environment to facilitate detailed processing of salient information (Corbett and Melcher, 2014b). Ensemble representations similarly affect how patients with focal attentional deficits perceive individual objects (e.g., Demeyere et al., 2008; Leib et al., 2012; Lanzoni et al., 2014; Pavlovskaya et al., 2015). In fact, no study has yet reported an instance where perceptual averaging can really be prevented, even when participants are explicitly instructed to do so (e.g., Oriet and Brand, 2013; see Dubé and Sekuler, 2015 for a recent review). Here, we further explored the fundamental, compulsory nature of statistical processing in a special population of highly functioning autistic individuals with noted behavioral differences that have been interpreted as suggesting that they may not incorporate perceptual averages into their visual representations of the external environment.

Autism Spectrum Disorder (ASD) is a developmental disorder broadly categorized along a continuum of severity by abnormalities in verbal and non-verbal communication, including fixating or restricting behaviors and interests to individual items and events, and deficits in adapting behavior to match the needs of different contexts (American Psychiatric Association [APA], 2013). ASD affects approximately 62 in 10,000 school-aged children worldwide (Elsabbagh et al., 2012). Although autism research is heavily focused on symptoms of social dysfunction, a number of studies have implicated sensory and motor systems as either part of the explanation of the social deficits or as parallel symptoms of ASD.

The visual system’s reliance on statistical regularities inherent in the external environment to guide perception may provide a powerful mechanism for coping with the potential sensory overload imposed by the massive amount of incoming visual information available in each glance (e.g., Corbett and Melcher, 2014b; Lanzoni et al., 2014), an ability that is thought to be a particularly vulnerable in ASD. Yet, given numerous findings suggesting an impaired ability in ASD to integrate individual pieces of visual information into coherent wholes, it is possible that these individuals do not perceptually average information. Testing whether this otherwise pervasive averaging process is manifest in ASD will further our understanding of how abstract statistical representations sub-serve human visual perception. Before describing the specific experiments we conducted to examine perceptual averaging in autistic individuals, we briefly review findings from relevant studies of ASD visual perception and several theories proposed to account for these findings.

### Visual Perception in ASD

Studies of visual perception in ASD typically use visual-spatial or syntactic versus semantic tasks to assess local and global processing abilities. There is convergent evidence from such studies that autistic individuals show enhanced local processing of features and fine detail. For example, individuals with ASD exhibit superior performance compared to controls on tasks that require speeded localization of individual objects embedded in a visually similar global context (e.g., Embedded Figures task: Shah and Frith, 1983; Happé, 1994; Jolliffe and Baron-Cohen, 1997, Visual search: Plaisted et al., 1998; O’Riordan et al., 2001), a superior ability to segment complex objects into constituent parts (Shah and Frith, 1993; Happé, 1994, 1999; Ehlers et al., 1997), and a preference for processing local features and details over global structure and form (e.g., Mottron et al., 1999).

**FIGURE 1:**
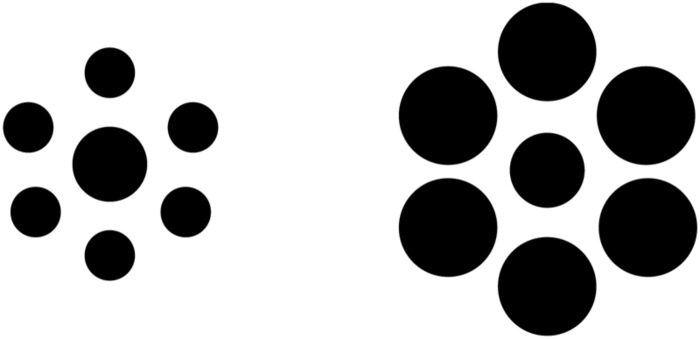
**Illustration of a typical Ebbinghaus display used in previous studies of size contrast illusions in ASD observers.** The central circles in each set are physically identical in size, but perceived as an inverse function of the sizes of the surrounding circles, such that the central circle surrounded by the set of smaller circles **(Left)** appears larger than the central circle surrounded by the larger set of circles **(Right)**.

The nature of global processing in autistic individuals is less clear. Several previous studies have found evidence that visual size contrast illusions persist in ASD using Ebbinghaus stimuli (**Figure 1**), similar to displays in the present investigation (e.g., Ropar and Mitchell, 1999, 2001; Hoy et al., 2004; c.f., Happé, 1996). Ebbinghaus and other low-level visual illusions are thought to arise from fundamental characteristics of perception, such that a lack of susceptibility is indicative of severe abnormalities in low-level visual processing (e.g., Bruce et al., 1996). Within this framework, ASD observers’ susceptibility to size contrast illusions suggest that this aspect of fundamental low-level perceptual processing remains intact.

Although results converge to suggest that at least some low-level global information is integrated in ASD vision, it is unclear whether ASD observers are able to rely on this type of information in the same manner as neurotypical observers. A number of studies of global visual perception in ASD participants point to differences in their default spatial scale of attention and associated deficits in broadening, shifting, or dividing attention. For example, whereas global information typically interferes with the processing of local information (e.g., Navon, 1977), Plaisted et al. (1999) confirmed that autistic observers also showed evidence of local interference on global letter identification in a Navon letter task when they had to divide their attention between the local and global letters on each trial (Mottron and Belleville, 1993), but not when they were instructed to selectively attend to only the global or local letter on each trial at the start of each block (Ozonoff et al., 1994, c.f., Rinehart et al., 2000). These results suggest that autism is associated with a deficit in spreading the focus of attention, or an “executive dysfunction” in inhibiting further processing of local information (e.g., Ozonoff et al., 1991; Hughes and Russell, 1993; Russell, 1997; Hill, 2004, 2008). Such hypersensitivity to local detail has also been used to explain previous findings that ASD observers fail to detect impossible figures (e.g., Mottron and Belleville, 1993; Mottron et al., 1999), and are slower and less accurate than controls to determine which of two stimuli presented in temporal succession is larger when the second stimulus is larger and they have to broaden their focus from the first smaller stimulus to the second larger stimulus (Mann and Walker, 2003).

There is also evidence that ASD involves an impaired ability to rely on top-down knowledge about the prototypical appearance of objects in the surrounding environment, such that autistic perception is less reliant on prior and contextual knowledge. For example, when Ropar and Mitchell (2002) presented autistic and control observers with a circle projected from an illuminated platform that tilted to produce an elliptical retinal image and asked them to adjust the size of a stimulus presented on a separate monitor to match the shape of the projected circle, both ASD and control participants exaggerated the circularity of the adjusted stimulus when it was surrounded by the context of two vertical lines, but ASD observers’ adjustments showed significantly less exaggeration than controls’ when the stimulus was presented in isolation. These patterns of results suggest that both groups were similarly affected by the vertical line cues to perspective, but ASD observers did not rely on the prior knowledge that the projected stimulus was actually a circle to the same extent as controls. A similar failure to rely on prior information has been linked to findings that ASD participants show reduced levels of perceptual adaptation to facial identity (e.g., Pellicano et al., 2007) and numerosity (Turi et al., 2015) compared to controls. A decreased reliance on prior knowledge could also account for findings that ASD observers fail to perceive impossible figures (e.g., Mottron and Belleville, 1993; Mottron et al., 1999). Furthermore, ASD observers have been reported not to show typical evidence of Gestalt grouping by proximity or similarity thought to arise from repeated exposure to canonical arrangements of objects in the surrounding environment (Brosnan et al., 2004).

Numerous theories have evolved to explain such findings regarding visual perception in autistic individuals. The Weak Central Coherence theory (WCC; Frith, 1989) is based on the idea that autistic individuals have increased focus on the smallest possible details, and therefore experience fragmented perception such that they cannot see past these details to construct a global “big picture.” The Enhanced Perceptual Function theory (EPF; Mottron and Burack, 2001) does not assume that global processing is impaired, but that individuals with ASD retain access to over-enhanced representations of low-level local and featural information rather than relying more on global, “gist” information over the course of information processing. More recent theories have emerged linking visual deficits in ASD to weaker influences of prior experience and related impairments in predictive processing abilities that result in more fleeting, veridical, and uncertain representations of the surrounding environment [e.g., Bayesian “hypo-priors,” Pellicano and Burr (2012); Predictive Impairment in Autism (PIA), Sinha et al., 2014]. Although a fully representative and exhaustive list of proposals that have been made to account for patterns of differences in ASD visual perception is beyond the scope of the current paper, we outlined this subset to provide support for the idea that perceptual averaging in ASD remains an open question, relevant to an active debate regarding theories of sensory processing in autism.

In the context of the present investigation, theories like the WCC that posit a deficit in constructing a coherent global representation would predict that ASD individuals are less likely to perceptually average information in sets of objects under any circumstances. Similarly, Bayesian “hypo-prior” and PIA theories predict that ASD observers are less likely to encode and/or incorporate prior knowledge about the mean size of objects into their psychophysical judgments of individual object size. Theories like the EPF that propose ASD is associated with atypical persistence of overly enhanced low-level local representations predict that ASD observers are only likely to perceptually average items when the task explicitly requires them to do so. In general, all of the theories outlined above predict that ASD participants would be more likely to encode and use the properties of specific objects than they are to be biased by the global average.

### Goal of the Present Study

In this initial investigation, we examined whether a group of high-functioning ASD observers showed evidence of perceptual averaging using simplified versions of two established discrimination and adaptation tasks, effects that we also replicated in a group of neurotypical observers. On the one hand, finding evidence of perceptual averaging in this special ASD population with noted visual and perceptual deficits would provide compelling support for the fundamental nature of perceptual averaging in human vision. On the other hand, finding no evidence of perceptual averaging in ASD observers would not rule out this possibility, but would support a foundation from which to test whether impairments in this fundamental process are linked to previously reported deficits in ASD visual perception.

## Experiment 1: Mean Versus Member

In Experiment 1, we measured participants’ accuracy in discriminating which of two test circles represented the mean size of a previously shown set of different-sized circles (*mean task*) and which of two test circles was a member of the previously shown set of circles (*member task*). Ariely’s (2001) original findings of superior representations of mean versus individual member size provided initial evidence of the visual system’s ability to represent the mean without needing to represent individual items. If autistic observers do not advantageously represent mean size, then they should perform with near chance accuracy in the mean task (e.g., Ariely, 2001; Corbett and Oriet, 2011). It is also possible that autistic observers may over-represent the local sizes of the individual circles in each set to the extent that they actually exhibit above chance performance in the member task. Alternatively, if ASD participants do advantageously represent mean size, then they should perform with superior accuracy in mean task.

### Methods

Written informed consent was obtained from all participants before beginning the experimental session, and the University of Trento’s local ethics committee had approved the procedures.

#### ASD Participants

Participants in the ASD group were recruited and tested at the Observation, Diagnosis, and Education Lab (ODFLab) at the Department of Psychology and Cognitive Science, University of Trento. The group was comprised of 12 young adults diagnosed with autism with high cognitive functions according to DSM-IV criteria by licensed clinical psychologists with extensive expertise in ASD. Participants were between the ages of 17 and 26 (mean age = 20.09 years, *SD* = 2.66 years, five females), all with normal or corrected-to-normal vision and hearing, and no history of other major psychiatric disorders or medical illness affecting visual or cognitive functioning. Participants had IQ scores within the normal range in the general population (mean IQ = 98.82, *SD* = 13.81), as measured by available scores for 11 participants on the Wechsler Intelligence Scale for Children-Revised (WISC-R; Wechsler, 1974), the Wechsler Intelligence Scale for Children-Third Edition (WISC-III; Wechsler, 1991), or the Wechsler Adult Intelligence Scale-Revised (WAIS-R; Wechsler, 1981). The IQ score for one participant previously diagnosed as high-functioning was not available. The testing situation included the presence of interns at the ODFLab (“experimenters”) who were familiar to the participants from other testing and therapy sessions. Both experimenters and participants were informed only of the general purposes and procedures of the experiments, and kept naïve to any specific hypotheses until data collection was completed for all participants.

#### Control Participants

We also tested a group of 12 neurotypical young adults between the ages of 19 and 26 (mean age = 22.33 years, *SD* = 2.42 years, seven females) with normal or corrected-to-normal vision, recruited from the student population of the University of Trento. These participants were tested under similar conditions as ASD participants, except that the ODFLab interns were not present and the experiments were conducted in a testing room in the psychophysical laboratories at the Center for Mind/Brain Science (CIMeC) at the University of Trento.

#### Task

**FIGURE 2:**
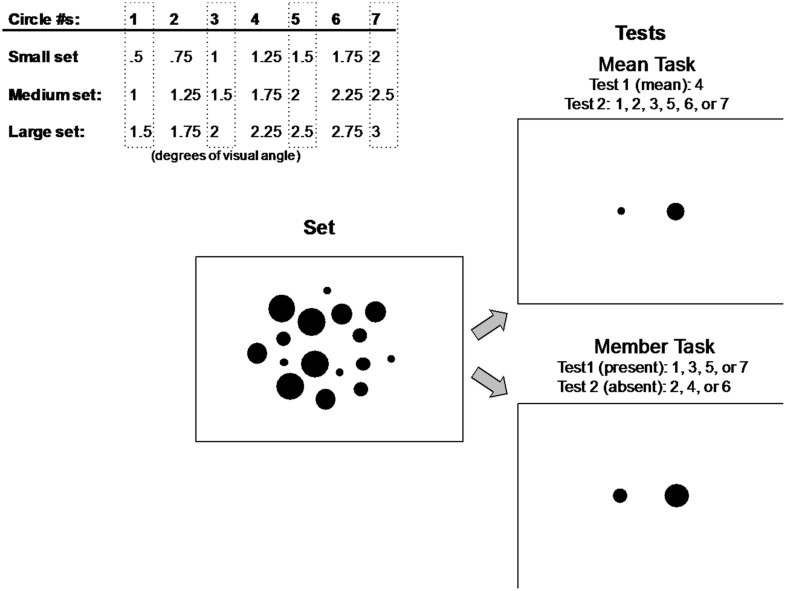
**Small, medium, and large circle sets used in Experiment 1 (top left).** Participants decided which of two test circles represented the mean (top right) or a member (bottom right) of a previously displayed set of circles (middle) composed of four repetitions of four circle sizes from one of three groups of seven circle sizes (top left). Circle sizes included in the set displays are surrounded by gray dashed rectangles and circles used in test displays are listed for each task.

On each trial, after viewing a briefly presented display of 16 filled circles, participants were presented with two test circles on the left and right sides of the screen (**Figure 2**). In the mean task, they were instructed to indicate which of the two test circles corresponded to the mean size of the previous set. In the member task, they indicated which circle was a member of the previous set. If the circle on the left/right was the mean/member, then they pressed the left/right arrow key on the computer keyboard. They were informed that only one of the two test circles was the mean/member, and to respond as quickly and accurately as possible on each trial.

#### Apparatus

Participants were tested individually in a semi-darkened room, seated in front of the center of the monitor, and restrained in a chin rest. In both testing environments, a Dell PC presented black circles against a gray (midway between black and white) background on 19-inch monitor with a vertical refresh rate of 60 Hz (1024 pixel × 768 pixel resolution) viewed from a fixed distance of 57 cm for all participants, such that 1° of visual angle subtended approximately 29 pixels. Responses were recorded using the computer keyboard. Matlab^®^ software (version 2009a) in conjunction with the Psychophysics Toolbox (Brainard, 1997; Pelli, 1997) controlled all the display, timing, and response functions.

#### Stimuli

One of the three groups of seven small, medium, or large circle sizes illustrated in **Figure 2** was used to construct the set and test displays on each trial in Experiment 1. These three different groups of sizes were used to prevent any effects of participants becoming overly familiar with a single group of sizes over the course of the experiment and perhaps basing their judgments on previously seen stimuli.

On each trial of Experiment 1, participants were presented with a display of 16 circles (**Figure 2**). The display of 16 circles was composed of four repetitions of the four different sizes in one of the three small, medium, or large groups of seven circles. These four sizes within each group were always circle #s 1, 3, 5, and 7, indicated in dashed rectangles in **Figure 2** (circle #s 2, 4, and 6 were never present in the set, and circle #4 represented the mean of each corresponding set). The positions of the 16 circles in each set were randomized on every trial. Each set of 16 circles was composed of two concentric rings, an inner ring of eight circles subtending 5° of visual angle, and an outer ring of eight circles subtending 9° of visual angle. Within each of the two rings, the eight circles were initially positioned at one of eight cardinal or 45° inter-cardinal locations, then jittered independently in the *x*- and *y*-directions by a random factor between -0.5° and +0.5° of visual angle on each trial. The positions of all 16 circles were restricted such that no individual circle was within 0.125° of any other circle in either the *x*- or *y*-direction.

The test displays consisted of two single circles presented at 2° eccentricity to the left and right of the horizontal meridian (**Figure 2**). In the *mean task*, one of these circles (left or right, determined randomly on each trial) corresponded to the mean size of the set (#4), which was never actually present in the set. The circle on the opposite side was one of the six other sizes in the group, regardless of whether it was present or not in the set, resulting in six possible test pairings. In the *member task*, one of the test circles was always a member present in the set (#s 1, 3, 5, or 7), and the opposite circle was never present in the set (#s 2, 4, or 6), resulting in 12 possible pairings of present/absent test circles.

#### Procedure

Each trial began with a 0.5° white fixation cross in the center of an otherwise blank gray screen. Participants were instructed to fixate the cross and press the spacebar to begin the trial. As soon as the spacebar was pressed, the fixation turned black, signaling that the trial was beginning. Participants remained fixating on the cross for 500 ms. Next, the set of 16 black circles was shown on the gray background (without the fixation cross) for 1000 ms. Immediately after the offset of the set of circles, two test circles were presented on a gray screen for 500 ms, followed by a blank gray screen except for a red fixation cross, signaling observers to make their response to the mean or member task.

The mean and member tasks were performed in separate blocks of trials. Each observer performed two blocks of each task for a total of four blocks of trials in Experiment 1. The order of the four blocks was counterbalanced over participants. In each task (mean or member), each block contained 36 trials and lasted less than 5 min. Each block of the mean task contained two trials with each of the 6 test pairings × 3 size groups, presented in pseudorandom order, for a total of 72 trials per point in the mean task. Each block of the member task contained one trial with each of the 12 test pairings × 3 size groups, presented in pseudorandom order, also for a total of 72 trials per point.

Before beginning the first block of each task, participants were given one block of 10 practice trials and written illustrated instructions, which were also re-presented at the start of every experimental block. The experimenter ensured all participants’ questions had been answered and they fully understood the task before advancing to the experimental blocks. All participants were informed of the length of each block, as well as the length of the entire experiment. They were also strongly encouraged not to move their heads or bodies for the entire duration of each experimental block.

#### Discrimination Task

To ensure that all participants could reliably discriminate between adjacent sizes of the seven different circles in each of the three sets of circles used in the experimental displays, they each completed one block of 18 trials in a discrimination task before beginning Experiment 1, comparing each adjacent-size circle pair (1 vs. 2, 2 vs. 3, 3 vs. 4, etc…) two times, with circle pairs presented in random order. On each trial, the circle pair was presented in the center of the screen for 500 ms, as outlined above for the test displays in the main Experiment 1, and participants had to judge which circle (left or right) was larger using the arrow keys. All subjects performed with 100% accuracy, indicating that they were all able to discriminate between each of the sizes tested in Experiment 1.

On all trials in the discrimination block and all experimental blocks in Experiments 1 and 2, the experimenter could press the ‘r’ key on the keyboard if the participant was not looking at the screen during the set or test displays, and the trial was stopped and redone at the end of the block. Only one trial was redone, for one ASD participant, because someone knocked on the door during testing.

Experiments 1 (including the discrimination block) and 2 were conducted during the same session with the same participants, experimenters, and apparatuses. The order of Experiments 1 and 2 was counterbalanced over participants in each ASD and control group. Participants always completed the discrimination block immediately prior to beginning Experiment 1. The total length of an entire experimental session was less than 45 min.

### Results

For both experiments in the present investigation, we compared performance within ASD and control groups separately. The data were analyzed in this manner to demonstrate evidence for the presence or absence of perceptual averaging in ASD, which is the main focus of the present investigation. Importantly, we used streamlined versions of Ariely’s (2001) original mean/member tasks (Experiment 1)^1^ and Corbett et al.’s (2012) adaptation task (Experiment 2)^2^ to obtain basic measures of perceptual averaging within the practical limitations imposed when testing ASD participants. We include data from the group of control participants in each experiment only to verify that our simplified tasks elicited the same type of perceptual effects previously reported for neurotypical observers. An investigation of any small differences in perceptual averaging between ASD and control groups would require a large sample size. Instead, we focused on a relatively homogenous group of high-functioning ASD participants who were willing and able to perform the tasks.

**FIGURE 3:**
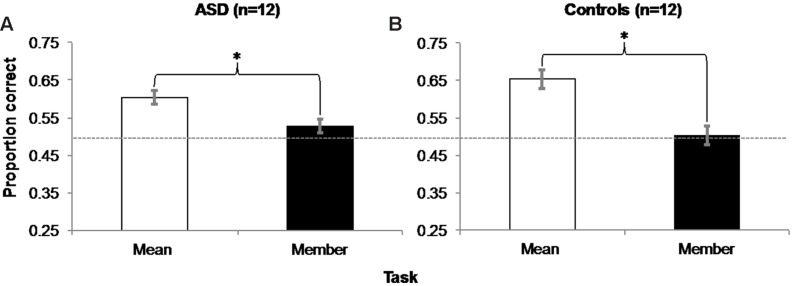
**Experiment 1 Results: Accuracy in the mean task was significantly greater than in the member task for both groups of ASD (A) and control (B) participants.** Error bars represent 95% within-subjects confidence intervals (Loftus and Masson, 1994) Asterisks represent significant main effects with *p* = 0.004 in the ASD group and *p* = 0.001 in the Control group. Dashed horizontal line represents 50% chance performance.

We analyzed the main effect of Task (mean or member) separately for ASD and control participant groups. To facilitate visual comparisons, results in the mean and member tasks are shown for both groups in **Figure 3**, and for individual participants in **Table 1**.

**Table 1:** Experiment 1 Individual Performance: Average proportion correct accuracy in the mean and member tasks for each of the 12 participants in the ASD (left) and control (right) groups, as well as grand-average accuracy (bold) and standard deviation (italic) for each task in each group.

	ASD (*n* = 12)		Control (*n* = 12)	
	Mean	Member	Mean	Member
	0.61	0.5	0.53	0.61
	0.56	0.5	0.78	0.44
	0.78	0.58	0.56	0.44
	0.58	0.5	0.58	0.44
	0.53	0.58	0.67	0.5
	0.58	0.47	0.58	0.44
	0.64	0.56	0.58	0.53
	0.5	0.42	0.64	0.56
	0.64	0.69	0.75	0.64
	0.61	0.56	0.72	0.5
	0.58	0.5	0.78	0.47
	0.64	0.47	0.67	0.47
**Average**	**0.6**	**0.52**	**0.65**	**0.5**
***SD***	*0.07*	*0.07*	*0.08*	*0.07*

#### ASD Participants

A within-subjects, repeated-measures ANOVA revealed a main effect of Task (Mean/Member) on accuracy [**Figure 3A**; *F*(1,11) = 13.091, *MSE* = 0.003, *p* = 0.004, η^2^ = 0.543]. Further one-sample *t*-tests confirmed that accuracy in the mean task (*M* = 0.604, *SD* = 0.071) was significantly different from chance [50% chance accuracy = 0.5 proportion correct; *t*(11) = 5.108, *SEM* = 0.02, *p* < 0.001, *d* = 0.426], but accuracy in the member task (*M* = 0.528, *SD* = 0.071) was not [*t*(11) = 1.344, *SEM* = 0.02, *p* = 0.206, *d* = 0.112]. To ensure that this accuracy difference was not due to a trade-off with reaction time (RT), we performed an ANOVA on RT between the two tasks, which was not significant (mean task: *M* = 739.53 ms, *SD* = 626.05 ms; member task: *M* = 849.87 ms, *SD* = 526.89 ms; *p* = 0.385).

#### Control Participants

A within-subjects, repeated-measures ANOVA also revealed a main effect of Task on control participants’ accuracy [**Figure 3B**; *F*(1,11) = 21.214, *MSE* = 0.006, *p* = 0.001, η^2^ = 0.659]. One sample *t*-tests also showed that accuracy in the mean task (*M* = 0.653, *SD* = 0.088) was significantly different from chance [*t*(11) = 5.984, *SEM* = 0.026, *p* < 0.001, *d* = 0.499], but accuracy in the member task (*M* = 0.503, *SD* = 0.069) was not [*t*(11) = 0.168, *SEM* = 0.02, *p* = 0.87, *d* = 0.014]. A follow-up ANOVA also showed no evidence of a significant difference in RT between tasks (mean task: *M* = 608.80 ms, *SD* = 437.35 ms; member task: *M* = 586.39 ms, *SD* = 413.47 ms; *p* = 0.623).

#### Overestimation of Mean Size

Previous studies of neurotypical observers have reported evidence of systematic overestimation of mean size (e.g., Chong and Treisman, 2003; Bauer, 2009; Corbett and Oriet, 2011; Corbett et al., 2012; Corbett and Melcher, 2014a; Corbett and Song, 2014). Given that ASD observers’ superior accuracy in the mean task demonstrates they indeed represent mean size, we next examined whether they also show evidence of an overestimation bias, such that they are more accurate in discriminating which of the two test circles was the mean size when the opposite test was smaller versus larger than the mean size test circle. This is a particularly interesting question given converging evidence suggesting that autistic individuals retain access to superior representations of features and fine details (Shah and Frith, 1983, 1993; Happé, 1994, 1999; Ehlers et al., 1997; Jolliffe and Baron-Cohen, 1997; Plaisted et al., 1998; O’Riordan et al., 2001). Along these lines, autistic participants might also retain a more precise representation of mean size, such that they are not susceptible to this bias characterized by asymmetric accuracy as a function of the difference in size between the mean and opposite test circle.

**FIGURE 4:**
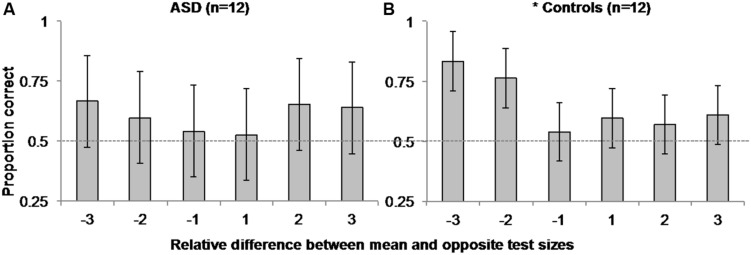
**Experiment 1 Mean Size Overestimation: Autistic participants (A) did not manifest the same positively skewed pattern of accuracy as controls (B) as a function of the relative difference in size between the opposite and mean test circles.** Error bars represent 95% within-subjects confidence intervals (Loftus and Masson, 1994). The asterisk beside control performance represents a significant main effect with *p* = 0.007 in a 1-way ANOVA comparing accuracy over relative step sizes. Dashed horizontal line represents 50% chance performance.

To explore this possibility, we separately examined ASD and control observers’ accuracy in the *mean task* as a function of the difference in the relative step size between the mean size test circle and the opposite test circle on each trial (i.e., the opposite test circle could be -3, -2, -1, 1, 2, or 3 steps from the mean test circle)^3^. We performed a within-subjects, repeated-measures ANOVA on accuracy between the six possible mean/opposite test circle pairings for each group. As expected, there was a main effect of the relative difference in opposite and mean test circle size [*F*(5,11) = 3.6, *MSE* = 0.046, *p* = 0.007, η^2^ = 0.247] accompanied by a significant linear trend [*F*(1,11) = 7.022, *MSE* = 0.066, *p* = 0.023, η^2^ = 0.39] on control participants’ accuracy. Taken with the positive skew illustrated in **Figure 4B**, these results suggest control participants were more accurate in discriminating which of the two test circles was the mean size when the opposite test was smaller than the mean versus when it was the same absolute difference in size but larger than the mean. However, there was neither evidence of a main effect [*F*(5,11) = 0.373, *MSE* = 0.111, *p* = 0.865, η^2^ = 0.033], nor of a linear trend [*F*(1,11) < 0.001, *MSE* = 0.366, *p* = 0.993, η^2^ < 0.001] of the relative difference in test circle size on ASD participants’ accuracy. There was also no corresponding positive skew apparent in **Figure 4A**.

### Discussion

Overall, participants were more accurate when deciding which of two test circles represented the mean size of the set of circles than they were at deciding which test circle was a member of the set shown on each trial. Furthermore, participants were better than chance in the mean judgment, but there was no evidence that they performed better than chance in the member task. Importantly, perfect accuracy in the initial discrimination task shows that all participants were able to perceive the difference in sizes of all adjacent circles used in the mean and member tasks of Experiment 1. Finally, the significant effect of the mean/member task in the control group confirmed that our simplified version of previously used perceptual averaging paradigms (e.g., Ariely, 2001; Corbett and Oriet, 2011) elicits expected effects in neurotypical observers. Taken together, these results suggest that autistic participants were able to represent the mean size of the set of circles even though they could not recall whether a given individual circle size was present in the set. These findings are in-line with previous findings of superior mean versus member performance in the more general population (Ariely, 2001; Corbett and Oriet, 2011).

Interestingly, ASD participants did not show the same pattern of mean size overestimation in the mean task observed here for controls and in several previous reports (Chong and Treisman, 2003; Bauer, 2009; Corbett and Oriet, 2011; Corbett et al., 2012; Corbett and Melcher, 2014a; Corbett and Song, 2014). We note that because we used two test circles in the mean task, it is possible that the pattern of results in control participants reflected an *underestimation* of individual size instead of an overestimation of mean size. However, in light of similar findings of mean size overestimation in neurotypical observers using a single test stimulus and the method of adjustment (Chong and Treisman, 2003; Bauer, 2009), it is more likely the present pattern of asymmetry in control observers’ accuracy as a function of the difference in size between the mean and opposite test circle reflects this typical overestimation of mean size. Although the null main effect of the difference in relative test circle size, and lack of a significant linear trend in ASD participants’ accuracy cannot directly be taken as evidence of a lack of overestimation bias in ASD observers, or that these observers are more accurate (show less overestimation of mean size) than controls, these findings do raise interesting and important questions about the precision of perceptual averaging in ASD and other special populations. Of greatest interest in the context present study, given previous findings that individuals with ASD are better at representing features and fine detail (Shah and Frith, 1983, 1993; Happé, 1994, 1999; Ehlers et al., 1997; Jolliffe and Baron-Cohen, 1997; Plaisted et al., 1998; O’Riordan et al., 2001), they may also retain a more precise representation of mean size such that they are less susceptible to this overestimation bias.

## E2: Adaptation to Mean Size

In Experiment 2, we tested whether participants showed evidence of perceptual adaptation to mean size. We used a simplified version of the paradigm originally used by Corbett et al. (2012) to demonstrate that neurotypical observers perceive individual test circle sizes as an inverse function of the average size of objects in preceding adaptor sets (a negative aftereffect of mean size adaptation). Participants adapted to two sets of test circles with different mean sizes, and then determined which of the two subsequently presented test circles was larger. Unbeknownst to participants, the two test circles were the same physical size on the majority of trials (only a handful of catch trials were present in each experimental block to ensure that participants did not become aware of this or frustrated by the lack of any perceived difference in any trial).

A negative aftereffect of mean size adaptation in ASD participants in Experiment 2 would offer further support for the proposal that mean size is encoded along a single visual dimension as a basic attribute (Corbett and Oriet, 2011; Corbett et al., 2012; Corbett and Melcher, 2014a; Corbett and Song, 2014). On the contrary, theories that propose individuals with autism do not construct global representations due to hypersensitivity to local detail (e.g., WCC: Frith, 1989), theories that propose autistics are better able to suppress the influence of global properties (e.g., EPF: Mottron and Burack, 2001) and only represent global properties when explicitly instructed to do so (Mottron et al., 1999; Plaisted et al., 1999; Rinehart et al., 2000), and theories that propose impairments in ASD are linked to deficits in encoding or incorporating prior information (e.g., Pellicano and Burr, 2012; Sinha et al., 2014) all suggest that ASD observers may not show evidence of such perceptual averaging adaptation aftereffects.

### Methods

The same groups of ASD and control observers performed Experiment 2 under the same experimental conditions as in Experiment 1.

#### Task

After adapting to two patches of heterogeneously sized circles on the either side of the screen, participants were presented with two single test circles and asked to judge which circle was larger (left or right) using the corresponding arrows on the computer keyboard. They were instructed to respond as quickly and accurately as possible and to guess if they were unsure.

#### Stimuli

**FIGURE 5:**
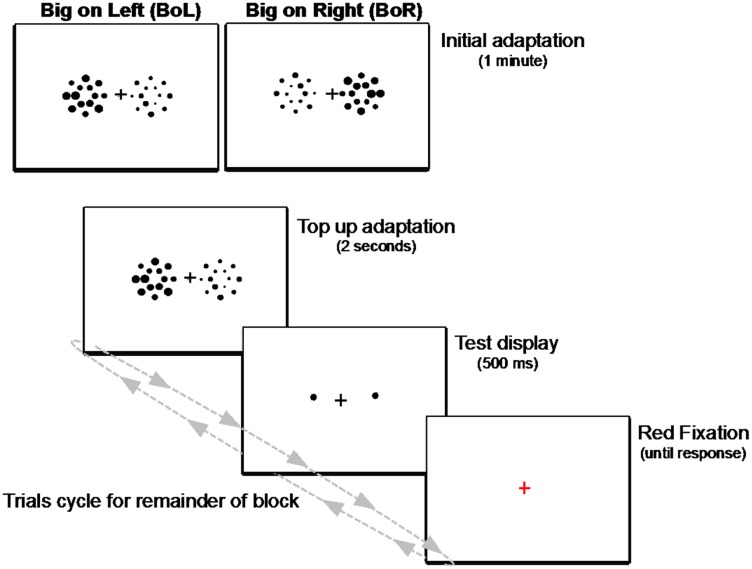
**Schematic illustration of the adaptation paradigm used in Experiment 2.** Each block began with an initial adaptation display of two side-by-side patches of big and small heterogeneously sized circles presented for 1 min. For the remainder of each block, trials cycled between a 2-s display of the circle patches to “top-up” adaptation on each trial, followed by a 500-ms test display of two single circles, then a red fixation prompt until response. Observers fixated the central cross during the entire block of trials, and their task was to determine which test circle (the left or right) was larger on every trial.

Adapting displays consisted of two sets of 14 circles (**Figure 5**). Each set was composed of two concentric rings, an inner ring of six circles subtending 2.5° of visual angle, and an outer ring of eight circles subtending 4.5° of visual angle. Circles in each set were positioned and jittered on each trial in the same manner as in Experiment 1.

The 14 circles in the smaller adapting set ranged in diameter from 0.5° to 1.15° in 0.05° steps, with a mean size of 0.825° of visual angle, and the 14 circles in the larger adapting set ranged in diameter from 0.85° to 1.5°, also in 0.05° steps, with a mean size of 1.175° of visual angle. Importantly, half of the circles in each set had the same seven diameters. We randomized the positions of the 14 individual circles in each adapting set on each trial to ensure that no location in either patch repeatedly contained a circle that was larger or smaller than any other circle in the set. Therefore, mean size (diameter) was the only constant difference between the two sets over the course of the experiment.

As in Experiment 1, the test displays consisted of two single circles, one on each side of the display. Unknown to subjects, in the majority of trials in each block (20), the two test circles were identical physical sizes (1° of visual angle). On the remaining five catch trials, one test circle (randomly selected as left or right) was either 0.8° or 1.2°, and appeared obviously smaller or larger than the opposite 1° test circle. We randomized the positions of the test circles within the two adapted regions from trial-to-trial, so that no given location in either adapted region was consistently probed, making it more likely that the mean size of the entire display of adapting circles was responsible for any observed effects on perceived size (e.g., Chong et al., 2008; Corbett and Oriet, 2011; Corbett et al., 2012).

As illustrated in **Figure 5**, each two-ringed adapting circle set was 8° of eccentricity from the center of the monitor, relative to the horizontal meridian. One test circle was presented in the center of each adapted region, with the *x*- and *y*-positions of each test circle jittered independently on each trial by a random factor between 0.5° and 1° of visual angle.

#### Procedure

Each participant performed a practice block of 15 trials, followed by two experimental blocks of 30 trials; one block with the larger mean size adapting set on the left side of the screen (Big on Left; BoL), and one block with the larger mean size on the right (Big on Right; BoR). Each block lasted less than 5 min. Within each block, the two test circles were physically equal in size on all but five (catch) trials. This resulted in 25 trials in each BoL and BoR Adapting condition for each participant. The order of Adapting conditions (BoL/BoR) was counterbalanced over observers. Practice blocks were also randomized over participants, such that there was no systematic relation between the big adapting side in the practice block and the order of the experimental (BoL/BoR) blocks. Participants were told that sometimes the two test circles might look equally sized, and to make their best guess if they were not sure which was larger. No other information was provided about the relative locations or sizes of the adapting displays. All participants received written illustrated instructions displayed on the experimental monitor, which were also re-presented at the start of every experimental block. The experimenter ensured that all participants’ questions had been answered and they fully understood the task before advancing to experimental blocks.

As shown in **Figure 5**, each block began with an initial adaptation phase, during which time participants fixated the central cross while viewing a display of the two side-by-side adapting patches for 1 minute. After this initial adaptation period, trials in each block cycled between a “top-up” adapting display presented for 2 s, to ensure adaptation was maintained throughout each block of trials, followed by a test display consisting of the two single circles and the fixation cross for 500 ms and, finally, a red fixation cross on an otherwise blank screen signaling participants to respond which of the two test circles was larger. The red fixation remained on the screen until participants responded, then the next trial’s top-up adaptation display was presented and the cycle repeated until the end of the block. Participants were instructed to remain fixated during the entire course of each experimental block, and the experimenter sat nearby and continuously watched them as they performed each trial to ensure they did not make any noticeable eye or head movements.

### Results

**FIGURE 6:**
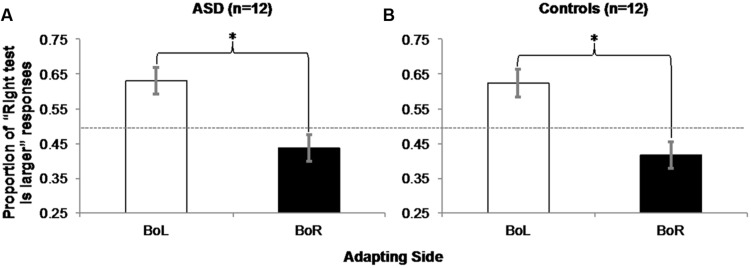
**Experiment 2 Results: ASD (A) and control (B) participants experienced a negative aftereffect of adaptation to mean size, such that they judged the right circle in a pair of two physically identical test circles as being larger significantly more often when they adapted to the big mean size patch of test circles on the left (and the small mean size patch on the right) than when they were adapted to the big mean size patch on the right (and the small mean size patch on the left).** Error bars represent 95% within-subjects confidence intervals (Loftus and Masson, 1994) Asterisks represent significant main effects with *p* = 0.002 in the ASD group and *p* = 0.001 in the Control group. Dashed horizontal line represents 50% chance performance.

For each participant in each BoL/BoR Adapting condition, we computed the average proportion of responses that the test circle on the right was larger (**Table 2**). All participants correctly chose the larger test circle in all catch trials, and these trials were excluded from further analysis. Two within-subjects, repeated-measures ANOVAs on the proportions of “right test is larger” responses in each Adapting condition (BoL/BoR) confirmed that both ASD [**Figure 6A**; *F*(1,11) = 16.432, *MSE* = 0.014, *p* = 0.002, η^2^ = 0.599] and control participants [**Figure 6B**; *F*(1,11) = 17.648, *MSE* = 0.015, *p* = 0.001, η^2^ = 0.616] experienced a significant negative adaptation aftereffect.

**Table 2:** Experiment 2 Individual Performance: Average proportion of “Right test circle is larger” responses in the BoL and BoR adapting conditions for each of the 12 participants in the ASD (left) and Control (right) groups, as well as grand-average accuracy (bold) and standard deviation (italic) for each condition in each group.

	ASD (*n* = 12)		Control (*n* = 12)	
	BoL	BoR	BoL	BoR
	0.52	0.48	0.84	0.72
	0.36	0.28	0.68	0.32
	0.72	0.28	0.44	0.24
	0.8	0.8	0.68	0.68
	0.52	0.44	0.68	0.52
	0.56	0.4	0.68	0.56
	0.84	0.36	0.56	0.16
	0.64	0.44	0.48	0.08
	0.48	0.24	0.68	0.52
	0.64	0.64	0.4	0.48
	0.48	0.12	0.92	0.44
	1	0.76	0.44	0.28
**Average**	**0.63**	**0.44**	**0.62**	**0.47**
***SD***	*0.18*	*0.21*	*0.16*	*0.2*

### Discussion

The results of Experiment 2 demonstrated that ASD participants’ perceptions of the sizes of individual test circles were biased by adaptation to mean size. This suggests that observers implicitly encoded the mean sizes of the sets of circles, even though this information was task irrelevant. In addition, the significant effect of Adapting condition in the control group confirms that our simplified version of Corbett et al.’s (2012) task is sensitive to mean size adaptation aftereffects previously demonstrated for neurotypical observers. Importantly, observers were never explicitly instructed to represent the mean size of the adapting patches, and accuracy in judging the sizes of the individual test circles was emphasized. This negative aftereffect of adaptation to mean size in ASD observers provides further support that mean size is encoded as a fundamental perceptual attribute, and is not predicted by previous proposals that individuals with autism do not construct global representations due to hypersensitivity to local detail (e.g., WCC: Frith, 1989), are not susceptible to size-contrast illusions (e.g., Happé, 1996), are better able to suppress the influence of global properties (e.g., EPF: Mottron and Burack, 2001), represent global properties only when explicitly instructed to do so (Mottron et al., 1999; Plaisted et al., 1999; Rinehart et al., 2000), or do not encode and/or incorporate prior knowledge about the mean size of objects into their psychophysical judgments of individual object size (e.g., Pellicano and Burr, 2012; Sinha et al., 2014).

## General Discussion

Two experiments examined whether the averaging process that has so far been found to be pervasive in visual perception is manifest in a special ASD population with noted behavioral differences that have been interpreted as suggesting that they may not incorporate such perceptual averages into their visual representations of the external environment. Across both experiments, both ASD and control participants showed behavioral effects of perceptual averaging, specifically the encoding of mean size. Most importantly, ASD participants performed with superior accuracy in the mean versus member task, indexing a perceptual advantage for mean versus individual representations (Experiment 1), and they were also susceptible to mean size adaptation (Experiment 2), providing further evidence for the fundamental and pervasive nature of statistical representations in visual perception (e.g., Ariely, 2001; Chong and Treisman, 2003, 2005; Corbett and Oriet, 2011; Corbett et al., 2012; Corbett and Melcher, 2014a; Dubé and Sekuler, 2015).

Notably, the significant effects of perceptual averaging observed for both groups were also reflected in individual performance. In Experiment 1, accuracy was higher in the mean versus member task for all but two ASD participants and one control participant (**Table 1**), and the effects of mean size adaptation on individuals’ size judgments in Experiment 2 are apparent for all but two other ASD participants and one other control participant who showed no difference between BoL and BoR adapting conditions, and one other control subject who showed an opposite response pattern between adapting conditions (**Table 2**). Therefore, our results confirm that these simplified versions of mean/member (Ariely, 2001; Corbett and Oriet, 2011) and mean size adaptation (Corbett et al., 2012) paradigms elicit perceptual averaging effects in both the ASD and control groups.

Although the present results support numerous proposals from previous studies of perceptual averaging, several of our findings do not align well with proposals from the literature regarding visual perception in ASD. For example, our findings that ASD observers represented mean size regardless of whether it was explicitly task relevant (Experiment 1, mean task) or irrelevant and even detrimental to the task (Experiment 2) provide strong support for claims that statistical summary representation is a fundamental, automatic, and unavoidable process (e.g., Ariely, 2001; Chong and Treisman, 2003, 2005; Corbett and Oriet, 2011; Corbett et al., 2012; Oriet and Brand, 2013; Corbett and Melcher, 2014a; Dubé and Sekuler, 2015). Yet these pervasive effects of mean size in ASD vision are difficult to reconcile with proposals that autistics will only represent global properties when explicitly instructed to do so (Mottron et al., 1999; Plaisted et al., 1999; Rinehart et al., 2000; Mottron and Burack, 2001), or when attention is directed globally versus locally (e.g., Plaisted et al., 1999). The persistent perceptual influences of mean size found here do fit well with previous proposals that even though items are not represented without focused local attention, mean size is represented both when attention is spread globally across an array of items and when it is focused locally to discriminate characteristics of individual items (e.g., Chong and Treisman, 2005). Along these lines, future studies of perceptual averaging in ASD could help to clarify whether autism is associated with deficits in spreading the focus of attention, and/or an “executive dysfunction” in inhibiting further processing of local information (e.g., Ozonoff et al., 1991; Hughes and Russell, 1993; Russell, 1997; Hill, 2004, 2008).

The present results demonstrating susceptibility to mean size adaptation also fit with previous findings that ASD observers are sensitive to size contrast illusions (e.g., Ropar and Mitchell, 1999, 2001; Hoy et al., 2004; c.f., Happé, 1996), providing converging evidence that this type of low level perceptual processing remains intact in ASD. It is also possible that the sizes of the individual test circles in Experiment 2 were processed as a function of top-down knowledge of depth cues (e.g., Ropar and Mitchell, 2002; Brosnan et al., 2004), such that stimuli presented to the region adapted to the smaller mean size patch were seen as larger because they were perceived to be farther away. Yet, such top-down influences cannot account for the findings of superior mean versus member performance in both groups of observers in Experiment 1. Along these lines, our results also have important implications for theories that posit impairments in predicting or integrating prior information in ASD (e.g., Pellicano and Burr, 2012; Sinha et al., 2014). For example, previous studies have reported that observers with ASD are less susceptible to adaptation to facial identity (Pellicano et al., 2007) and numerosity (Turi et al., 2015). However, the present results demonstrating susceptibility to mean size adaptation strongly suggest that ASD observers nonetheless rely on statistical redundancies in the external environment to some extent. As biases to represent central tendencies in visual information are advantageous for reducing variability and improving the reliability of perception, future studies of perceptual averaging in ASD have great potential to inform the development of techniques for reducing and coping with sensory uncertainty.

Although our findings converge to support the hypothesized fundamental nature of perceptual averaging in vision, there are several important considerations in interpreting the present results. As we have noted, our sample size was limited to a fairly homogenous group of 12 individuals diagnosed with highly functioning ASD, limiting us from drawing broader statistical comparisons between the two groups of ASD and control participants. In addition, we did not measure IQ in control observers under the assumption that these students from the University of Trento represented a sample of the general population with IQ scores roughly normally distributed in the same manner as the IQs of highly functioning ASD participants. However, it is possible that differences in IQ are also involved in the present findings. For example, it is possible that the university students tested in the control group may have had a slightly higher average IQ, or that ASD participants may have scored higher in performance IQ measures than controls due to compensatory mechanisms relating to stronger visuo-spatial abilities. Although these issues were outside the scope of our initial investigation of whether there is any evidence of perceptual averaging in ASD, they do provide strong motivation for future research using similar paradigms to quantify any systematic differences related to perceptual averaging that may be manifest in larger samples of ASD and control participants, perhaps by testing participants from the general public and correlating their performance on standardized measures of ASD with their performance in tasks such as the present mean/member and adaptation tasks (e.g., Lowe et al., 2016).

Even though the present experiments were not designed to address potential differences in the perceptual averaging abilities of ASD and neurotypical observers, several aspects of our results support the merit of such further investigations with a much larger sample size. Whereas perceptual averaging effects were present for almost all participants in the present investigation, in Experiment 1, the relative effect of mean/member task on ASD participants’ accuracy was about of half the size of the corresponding effect on control participants’ accuracy (15%), and ASD observers did not show evidence of typical patterns of mean size overestimation exhibited by control participants. Although these results are not entirely inconsistent with predictions from the WCC Theory (Frith, 1989) that ASD observers will show decreased reliance on perceptual averaging and more precise local representations due to weak (but not absent) central coherence and hypersensitivity to local detail, these results also raise the interesting possibility that individuals with ASD may actually be more accurate at encoding mean versus individual size. Along these lines, both groups performed poorly in the member task in Experiment 1. Based on previous studies showing superior performance in ASD versus control observers on tasks involving local details (Shah and Frith, 1983, 1993; Happé, 1994, 1999; Ehlers et al., 1997; Jolliffe and Baron-Cohen, 1997; Plaisted et al., 1998; O’Riordan et al., 2001), we might have expected above chance performance in the member task for the ASD group. Of course it is possible that with a smaller set size some differences may have emerged, and/or that the member task was too difficult/computationally demanding to allow for an effective assessment of the precision of individual size representations, even though only four different sizes were ever presented within a given set. It is also important to point out that higher accuracy in the mean task cannot be taken as evidence that the mean was represented more precisely than could be expected by averaging the representations of individual member sizes. In any case, these findings point to the need for future studies measuring the precision of individual and average representations in advancing our understanding of visual perception in ASD.

In Experiment 2, the magnitude of mean size adaptation was similar for ASD (19.3%, η^2^ = 0.599) and controls (20.67%, η^2^ = 0.616), contrary to previous reports of weaker adaptation to individual facial identity (Pellicano et al., 2007) and numerosity (Turi et al., 2015) in ASD. Although null results of statistical comparisons between the groups of 12 ASD and 12 control participants in the present investigation could not be meaningfully interpreted, these comparable magnitudes of mean size adaptation suggest that significant between-group differences such as those recently reported by Turi et al. (2015) for numerosity adaptation with groups of 16 ASD and 18 control participants are not likely to emerge for mean size adaptation with larger groups of participants. In light of our present findings of mean size adaptation in ASD, a particularly interesting avenue of future research involves examining whether perceptual averaging in ASD also extends to socially meaningful stimuli such as faces.

Finally, previous studies have measured susceptibility to the Ebbinghaus illusion in ASD observers using both verbal reports (Happé, 1996; Hoy et al., 2004) and manual estimations (Ropar and Mitchell, 1999, 2001). There is still a controversy regarding whether and how size-contrast effects such as the Ebbinghaus illusion are manifest in neurotypical perception and action. On the one hand, numerous studies report size-contrast effects on perception but not action (e.g., Aglioti et al., 1995; Haffenden and Goodale, 1998; Marotta et al., 1998; Haffenden et al., 2001). On the other hand, there are numerous other studies that do not provide evidence for such dissociation with similar size-contrast effects across perception and action tasks (e.g., Pavani et al., 1999; Van Donkelaar, 1999; Franz et al., 2000; Glover and Dixon, 2001; Kopiske et al., 2016). Along these lines, Corbett and Song (2014) demonstrated that visually guided actions directed to test circles presented in regions adapted to large/small mean sized displays were initially biased in-line with a persistent perceptual aftereffect. Theories such as the “dorsal stream vulnerability” hypothesis propose that impairments in dorsal mechanisms responsible for visually guided actions and the encoding of contour and global motion (Spencer et al., 2000; Braddick et al., 2003; Atkinson and Braddick, 2005), and/or low-level impairments in magnocellular visual processing (e.g., Milne et al., 2002) are involved in purported autistic global processing deficits (but see Dakin and Frith, 2005). Therefore, it would be interesting in future research to examine whether ASD observers’ visually guided actions to stimuli presented in regions adapted to large/small mean sizes are also biased in-line with the perceptual aftereffect.

Overall, the persistence of perceptual averaging in this special population underscores both the fundamental nature of statistical representations in vision, and the need for future research to advance our limited understandings of the mechanisms involved in statistical representations and in differences found in perception for ASD. The most important implication of the present findings is that at least some forms of global statistical processing are automatic and intact in a group of high-functioning ASD participants. Starting with the idea that the “gist” of the scene is represented automatically in both neurotypical and autistic persons, there is more room for training individuals with ASD to more effectively use their intact representations of global information than if such visual statistics were not encoded.

## Author Contributions

This research was supported by the Autonomous Province of Trento through the call “Grandi Progetti 2012”, project “Characterizing and improving brain mechanisms of attention – ATTEND”, and the Fondazione Cassa di Risparmio di Trento e Rovereto.

## Conflict of Interest Statement

The authors declare that the research was conducted in the absence of any commercial or financial relationships that could be construed as a potential conflict of interest.

## Footnotes

1 Ariely (2001) originally used different set sizes of 4, 8, 12, and 16 circles and a broad range of test circle sizes to measure member identification and mean discrimination in separate experiments. Member identification was measured in both a yes/no paradigm with a single test stimulus which participants judged as either matching or not matching one of the items in a previously presented set of circles, and a two-alternative forced choice (2-AFC) paradigm where participants had to select which of two test stimuli matched a member of the previously shown set. Mean discrimination performance was measured with a single test circle which participants judged as larger or smaller than the mean size of the set of previously shown circles.

2 In Corbett et al.’s (2012), we used the method of constant stimuli to measure the difference in the physical size between two test circles necessary to perceive them as the same size when observers were adapted to a large mean size patch of circles on one half of the display and a small means size patch on the opposite side.

3 Note that it was not possible to make similar comparisons about the accuracy of judgments in the *member* task of Experiment 1, in which one test circle was a member of the set and the opposite test circle was not a member of the set. Although the non-member test circle could be the mean or a non-mean size that was not present, and also could be several different step sizes from the member test circle, the non-member test circle could also be various step sizes from other members of the set that were not represented by the member.
